# Domain Correction Based on Kernel Transformation for Drift Compensation in the E-Nose System

**DOI:** 10.3390/s18103209

**Published:** 2018-09-23

**Authors:** Yang Tao, Juan Xu, Zhifang Liang, Lian Xiong, Haocheng Yang

**Affiliations:** School of Communication and Information Engineering, Chongqing University of Posts and Telecommunications, Chongqing 400065, China; S160131179@stu.cqupt.edu.cn (J.X.); xionglian@cqupt.edu.cn (L.X.); S170101083@stu.cqupt.edu.cn (H.Y.)

**Keywords:** drift compensation, transfer learning, domain correction, electronic nose

## Abstract

This paper proposes a way for drift compensation in electronic noses (e-nose) that often suffers from uncertain and unpredictable sensor drift. Traditional machine learning methods for odor recognition require consistent data distribution, which makes the model trained with previous data less generalized. In the actual application scenario, the data collected previously and the data collected later may have different data distributions due to the sensor drift. If the dataset without sensor drift is treated as a source domain and the dataset with sensor drift as a target domain, a domain correction based on kernel transformation (DCKT) method is proposed to compensate the sensor drift. The proposed method makes the distribution consistency of two domains greatly improved through mapping to a high-dimensional reproducing kernel space and reducing the domain distance. A public benchmark sensor drift dataset is used to verify the effectiveness and efficiency of the proposed DCKT method. The experimental result shows that the proposed method yields the highest average accuracies compared to other considered methods.

## 1. Introduction

The electronic nose (e-nose) is an intelligent system consisting of a set of sensors combined with corresponding pattern recognition algorithms to identify gases. When the gas enters the detection chamber, the gas sensor will generate a transient response signal. Then, the pattern recognition algorithm is applied to identify the gas based on the sensor response [1,2]. The e-nose has developed rapidly in the past 20 years and has been used for detecting and classifying the perfumes, black tea, wine, fruits, bacteria, coffee, etc. Laref et al. [3], Modak et al. [4], Paknahad et al. [5] and Chen et al. [6] used e-noses to monitor gas concentration, classify black tea based on aroma profile, analyze the wine quality, and classify fruit maturity. Liang et al. [7] used an e-nose to detect bacteria in the wound infection.

Many pattern recognition algorithms for classification and regression of e-noses have been proposed [8,9,10,11]. Neural networks play an important role in pattern recognition algorithms. Rumelhart et al. [12] proposed the error back propagation (BP) algorithm. Simplified fuzzy ARTMAP (SFAM) is a simplification based on the ARTMAP network foundation of adaptive resonance theory [13]. Support vector machine (SVM) is a pattern recognition method based on statistical learning theory [14]. Extreme learning machines (ELM) are an easy-to-use and effective learning algorithm to solve single hidden-layer feed forward neural networks [15]. ELM is widely used in classification and regression.

These algorithms are classical classification algorithms in machine learning, and are often used to identify the gas in e-nose systems. Zhang et al. [16] proposed a hybrid linear DA (LDA)-based SVM method to classify six air pollutants. Wolfrum et al. [17] used e-noses to detect volatile organic compounds in the air. However, as time goes on, the performance of prediction model trained by the original data is getting worse and worse. It is due to the sensor drift that makes the distribution of obtained data differ from the training data. The sensor drift is caused by thermo mechanical fatigue, heater drift, the influence of open environment, temperature, humidity, etc., and it is irregular and uncertain. Due to the difference of the data distribution, the traditional machine methods are not applicable. However, the transfer learning is an appropriate method for drift compensation. It tries to transfer the knowledge from some previous tasks to a target task where the latter has fewer training data [18].

Motivated by the idea of transfer learning, we focus on the compensation of sensor drift and try to improve the prediction accuracy from the perspective of data distribution. Usually, we refer to the e-nose data without sensor drift as source domain; the data with sensor drift as target domain. Because the traditional machine learning algorithms require that the data distribution between the source and target data be the same, the models trained by source domain data cannot be used directly on target domain for prediction. This limits the application and development of the e-nose, so the focus of this paper is to suppress drift and improve recognition accuracy from the perspective of domain correction.

**Figure 1:**
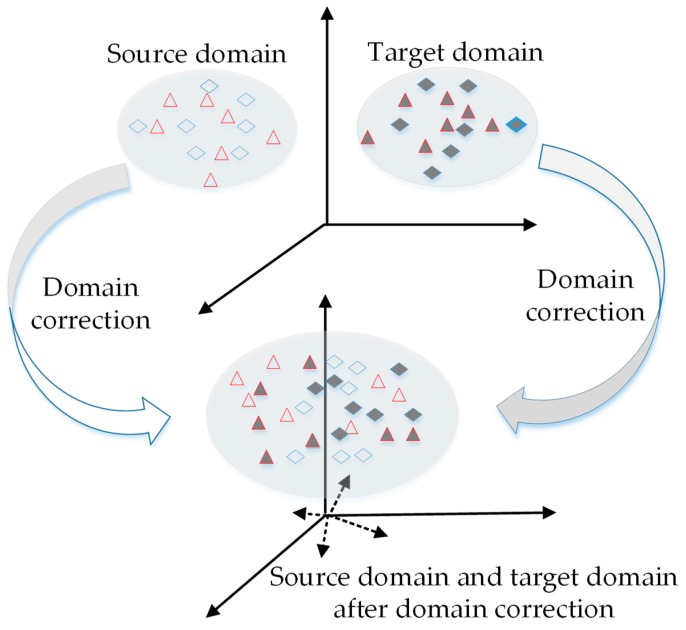
Schematic diagram of the proposed DCKT method.

From the above, the traditional pattern recognition algorithms are not always suitable for the e-nose. The model trained on source domain cannot be used directly on target domain because the distribution of data features between source domain and target domain are different. Therefore, we propose a domain correction based on kernel transformation (DCKT) method to solve this issue, which perform domain correction on source and target domain data. After domain correction, the data distribution difference between source domain and target domain can be significantly reduced and the data properties are preserved. The basic idea of DCKT is illustrated in Figure 1. Then, with the proposed DCKT method, we are able to achieve better accuracy and improve the antidrift performance of predictive models.

The rest of this paper is organized as follows. Section 2 presents the related work of sensor drift compensation and transfer learning. The proposed method DCKT for drift compensation is proposed in Section 3. The experiments and results have been discussed in Section 4. Finally, Section 5 concludes this paper.

## 2. Related Work

### 2.1. Sensor Drift Compensation

Drift is an unavoidable problem in e-noses. Due to the properties of a gas sensor, the output signal will fluctuate with environmental factors, self-aging, and other causes, which is called sensor drift. The essence of solving the drift problem lies in improving the reliability and stability of the gas sensor. However, this requires breakthroughs in many aspects, such as physics, chemistry, materials, and manufacturing processes, which are time consuming and costly. On the other hand, signal processing methods which are low-cost can be used to suppress the sensor drift. This paper mainly studies the drift problem of E-nose system from the perspective of signal processing.

According to the characteristics of the drift, we can divide the sensor drift into long-term drift and short-term drift. For the short-term drift, there is a certain functional relationship between the fluctuation amplitude and environmental factors (such as temperature, humidity and pressure), and it is generally reversible. Therefore, for short-term drift, the approximate functional relationship can generally be found through mathematical methods such as neural network and data fitting, and then the drift effect caused by environmental factors can be eliminated. Long-term drift refers to the changes in sensor response patterns over time. It is hard to find an exact function to describe the long-term drift of sensors because of the slow random fluctuation on sensor response. Therefore, using the functional relationship to eliminate the drift of the sensor is not a good choice.

The performance of the e-nose largely depends on the stability and sensitivity of the sensors. In particular, the performance of the trained discriminant model will deteriorate when the e-nose is subject to drift. Thus, the drift compensation should be taken into consideration. There are multiple works suggest that compensation methods can be defined as five categories [19]. They are: (1) sensor signal preprocessing methods, such as baseline manipulation [20], frequency domain filtering; (2) Traditional sensor response correction methods [21], such as principal component analysis (PCA) [22], partial least squares (PLS) [22], orthogonal signal correction (OSC) [23,24], etc.; (3) adaptive estimation methods, such as the adaptive self-organizing map (SOM) [25], domain adaption methods [26], etc.; (4) conditional machine learning method; (5) methods to improve the generalization of the classifier, such as the kernel fuzzy C-means clustering method, kernel fuzzy support vector machine (K-FSVM) method [27], ensemble of classifiers methods [28], etc.

The above methods have reduced the effects of drift in e-nose to a certain extent, but they are all focused on one data domain which leads to a weak generalization of newly collected data. The DCKT methods proposed in this paper is completely different from the previous method. DCKT aims at domain correction of the two domains, which solves the poor generalization of newly collected data.

### 2.2. Transfer Learning

Traditional machine learning usually has two basic assumptions: (1) training samples and test samples satisfy independent identical distribution; (2) training samples should be sufficient. However, it is difficult to satisfy these two basic assumptions in real life sometimes. The sensor drift makes the obtained data do not satisfy these two assumptions, and the models trained by the data obtained in last month are often difficult to apply to predict in the next month. In order to solve this problem, transfer learning that caused widely concern can be considered [18].

**Figure 2:**
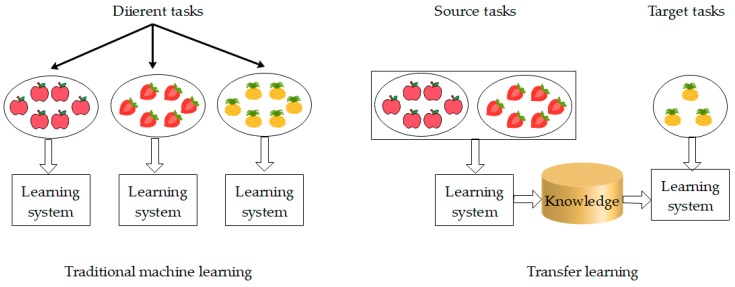
Difference between traditional machine learning and transfer learning.

The problem that transfer learning solves is: there is little or no label data in the target task. But the source task has sufficient data which are related to the target task but out of date (i.e., there is a difference between the target task data and the source task data). In other words, transfer learning can apply previously learned knowledge to new learning tasks, which is also the difference from traditional machine learning. Figure 2 shows the difference between traditional machine learning and transfer learning.

From Figure 2, it can be seen that traditional machine learning attempts to learn each task from its own training sample, while transfer learning stores knowledge gained from solving one problem and applies it to a different but related problem. For example, if one learns to ride a bicycle, he can quickly learn to ride a motorcycle because the two tasks are related.

The distribution of data obtained from the e-nose which suffers from sensor drift is different from the distribution of standard data, and it is one of the scenarios that transfer learning solves. Many researches have paid much more effort on drift compensation with the idea of transfer learning. Yan et al. [29] proposed a drift correction automatic encoder (DCAE) to solve the drift problem in e-noses. Zhang et al. proposed a domain adaptation extreme learning machine framework (DAELM) to compensate drift in e-noses [30]. Yan et al. proposed a transfer sample-based coupled task learning (TCTL) framework, which solves the problem of sensor drift [31]. All of these methods are from the perspective of transfer learning. It is worthwhile to study the drift compensation in e-noses with the idea of transfer learning.

## 3. Domain Correction Based on Kernel Transformation (DCKT)

### 3.1. Notation

In this paper, the source and target domains are denoted by the subscript “*S*” and “*T*”. The source domain sample set is denoted as $X_{S}=[x_{S}^{1},\cdots ,x_{S}^{N_{S}}]\in {ℜ}^{D\times N_{S}}$, and the corresponding true values are $y_{S}=[y_{S}^{1},\cdots ,y_{S}^{N_{S}}]$, which are treat as the training sample set. The target domain sample set is denoted as $X_{T}=[x_{T}^{1},\cdots ,x_{T}^{N_{T}}]\in {ℜ}^{D\times N_{T}}$, where *D* is the dimensionality, and $N_{S}$ and $N_{T}$ are the number of samples in source and target domains, respectively.

### 3.2. Domain Correction Based on Kernel Transformation

To make the feature distribution difference between the source and target domain data smaller, the source domain and the target domain are mapped to a high-dimensional reproducing kernel Hilbert space through a nonlinear mapping [32]. In this space, the feature distributions of source domain and target domain can be considered as consistent, i.e., the distance between the source and the target domains is minimized. At the same time, data properties are preserved to the utmost extent.

The objective function that minimizes the distance between the source and target domains after mapping is as follows:

(1) $$\underset{}{\min}\mathrm{dist}(X^{\prime }{}_{S},X^{\prime }{}_{T})={\left\|\frac{1}{N_{S}}\sum_{i=1}^{N_{S}}\phi (x_{Si})-\frac{1}{N_{T}}\sum_{i=1}^{N_{T}}\phi (x_{Ti})\right\|}_{H}^{2}\;$$

And then introduce the kernel matrix **K**, which constructed as follows:

(2) $$K=\left[\begin{array}{cc} K_{S,S} & K_{S,T}\\ K_{T,S} & K_{T,T} \end{array}\right]\in {\mathbb{R}}^{\left(N_{S}+N_{T})\times (N_{S}+N_{T}\right)}\;$$

**L** is the distribution difference matrix whose elements are:

(3) $$L_{ij}=\{\begin{array}{c} \frac{1}{N_{S}\cdot N_{S}}\;(x_{i},x_{j}\in X_{S})\\ \frac{1}{N_{T}\cdot N_{T}}\;(x_{i},x_{j}\in X_{T})\\ \frac{-1}{N_{S}\cdot N_{T}}\;(\mathrm{otherwise}) \end{array}\;$$

After introducing Equations (2) and (3), the objective function can be written as:

(4) $$\underset{}{\max}\mathrm{tr}(KL)-\lambda \mathrm{tr}(K)\;$$

The distance between distributions of different domains is minimized in the first term, while the variance in the feature space is maximized in the second term, and λ≥0 is a tradeoff parameter.

To solve this SDP problem which with high computational burden, a dimensionality reduction method for domain adaptation is proposed [32]. A matrix **P** is introduced to transfer the empirical kernel map features to an *m*-dimensional space. The kernel learning problem becomes

(5) $$\begin{array}{l} \underset{}{\min}\mathrm{tr}(P^{T}KLKP)+\mu \mathrm{tr}(P^{T}P)\\ s.t.\;P^{T}KHKP=I_{m} \end{array}$$

where μ>0 is the penalty coefficient, $I_{m}\in {\mathbb{R}}^{m\times m}$ is the *m*-dimensional identity matrix, **H** matrix is a central matrix which can be expressed as:

(6) $$H=I_{N_{S}+N_{T}}-(\frac{1}{N_{S}}+N_{T})11^{T}.$$

And $\mathrm{tr}(P^{T}KLKP)$ is the maximum mean discrepancy (MMD) between the empirical means of the two domains $X_{S}^{\prime }$ and $X_{T}^{\prime }$, and a regularization term $\mathrm{tr}(P^{T}P)$ used to control the complexity of **P**. The purpose of the constraints is to maintain data properties of source domain and target domain.

Equation (5) can be simplified as:

(7) $$\underset{P}{\max}\mathrm{tr}({(P^{T}(KLK+\mu I)P)}^{-1}P^{T}KHKP)$$

Similar to the kernel Fisher discriminant analysis [33], the solution of P in Equation (7) is the *m* smallest eigenvectors of ${\left(KLK+\mu I\right)}^{-1}KHK$, where $m\leq N_{S}+N_{T}+1$, represented by:

(8) $$P*=\left[p_{1},p_{2},\ldots ,p_{m}\right]\;$$

For easy implementation, the proposed DCKT algorithm is summarized in Algorithm 1. 

**Algorithm 1** DCKT**Input**: Source data $X_{S}$, target data $X_{T}$, source label $y_{S}$, regularization coefficients μ, and dimension *m*: **Procedure:** 1. Construct the kernel matrix **K** from ${\left\{x_{S}^{i}\right\}}_{i=1}^{N_{S}}$ and ${\left\{x_{T}^{i}\right\}}_{i=1}^{N_{T}}$ via (2), matric **L** via (3), and centering matric **H** via (6); 2. Solve the eigendecomposition of ${\left(KLK+\mu I\right)}^{-1}KHK$; 3. Build **P** by *m* smallest eigenvectors via (8); 4. Compute the mapped source domain data $X_{S}^{\prime }=P^{T}X_{S}$; 5. Compute the mapped target domain data $X_{T}^{\prime }=P^{T}X_{T}$; 6. Train the SVM classifier with $\left\{X_{S}^{\prime },y_{S}\right\}$, and predict the odor label of $X_{T}^{\prime }$;**Output:** The classification results of target data.

The proposed DCKT is used to perform domain correction on source and target domains. After domain correcting, the distance between source domain and target domain become closer, and the data properties of domains are preserved. The source domain data $X_{S}^{\prime }=P^{T}X_{S}$ and target domain data $X_{T}^{\prime }=P^{T}X_{T}$ after mapping can be used in support vector machine (SVM) for train and test.

## 4. Experimental and Performance Evaluation

This section will present the experimental results to demonstrate the effectiveness of the DCKT algorithm proposed in this paper.

### 4.1. Experimental Data

In this paper, a verification experiment of the proposed DCKT method is carried out using the sensor drift benchmark dataset of up to three years, from January 2008 to February 2011, collected by the e-nose from Vergara et al. [28] in University of California, San Diego (UCSD). The e-nose system is exposed to six kinds of gaseous substances, such as acetone, acetaldehyde, ethanol, ethylene, ammonia, and toluene at different concentration levels. This dataset contains 13,910 samples, which are divided into 10 batches of time series to make the number of measurements is as uniformly distributed as possible. The details of the number of samples for each category of each batch are shown in Table 1. The e-nose system consists of 16 gas sensors, each of which extracts eight features. Therefore, a 128-dimensional feature vectors (16 × 8) for each sample is formulated. Vergara et al. [34] introduced the details of feature extraction. In order to reduce the dimensional difference between the dimensions, the data is centralized.

**Figure 3:**
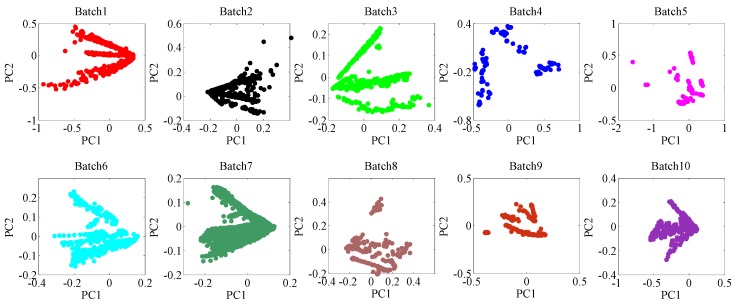
Principal components (PC1 vs. PC2) of the raw data of 10 batches using PCA (i.e., the 2-D subspace distribution of 10 batches, respectively), from which the significant changes of data space distribution caused by drift can be observed.

In order to observe and analyze the distribution of these 10 batches of datasets intuitively, the principal component analysis (PCA) is performed on these 10 batches of datasets, and the scatter points are shown in Figure 3. As time goes by, the 2-D subspace distribution between first batch (Batch1) and other batches is significantly biased due to the drift.

Data with different distributions is called heterogeneous data, and this dataset has a certain difference in the distribution of each batch. Therefore, for each batch of datasets, other batches of datasets are heterogeneous data. The data of the Batch 1 is considered as data before drift (i.e., source domain), and as a training sample set. However, other batches of datasets have drifted compared to Batch 1 because their distribution has significant difference, and can be considered as target domain. If the traditional classifier trained by the Batch 1 is used to classify the other batches, the recognition performance of the classifier will decline. Because it violates the basic assumptions of machine learning: training samples and test samples should have the same or similar probability distribution (independent identical distribution).

To demonstrate the effectiveness of the proposed DCKT method for distribution alignment and domain correction, the qualitative and quantitative experiments are presented, respectively.

### 4.2. Qualitative Result

**Figure 4:**
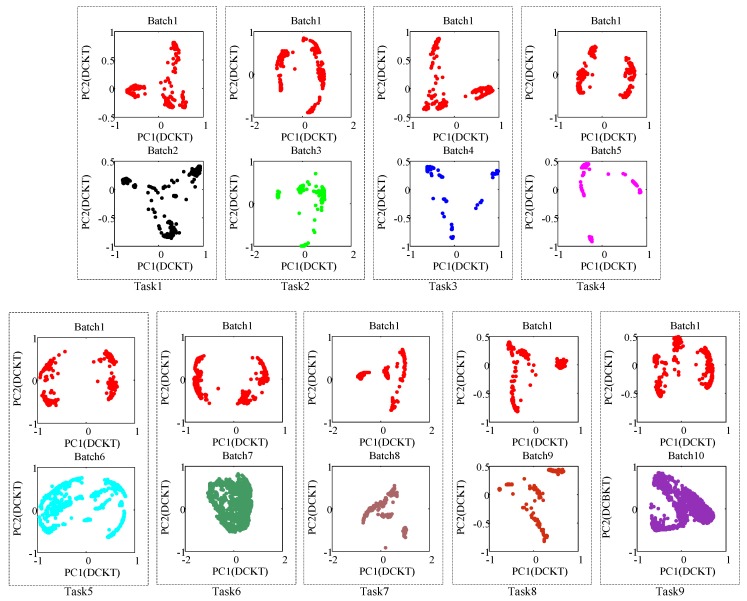
Principal components (PC1 vs. PC2) of the source domain and the target domain after DCKT (i.e., the 2-D subspace distribution of 9 tasks, respectively). In each task, the top picture is the Batch 1 (source domain) after DCKT and the picture below is the Batch *i* (target domain) after DCKT, from it we can find that the distribution of source domain and target domain is more similar after DCKT.

In this paper, the dataset of Batch 1 is treated as the source domain, and the dataset of Batch *i* (*i* = 2, 3, ..., 10) is treated as the target domain. Batch 1 and Batch *i* (*i* = 2, 3, ..., 10) are paired into Task (*i* − 1). Therefor, 9 pairwise principal component scatter points after using DCKT are shown in Figure 4. From the PCA scatter points, we can see that the probability distribution between source and target domains of each task has been greatly closer.

### 4.3. Quantitative Result

The purpose of the DCKT method proposed in this paper is to improve the performance of the classifier. Therefore, the recognition accuracy of six classes on each batch is used as a criterion. The recognition accuracy *RC* can be defined as follows:

(9) $$RC=\frac{N_{\left(y_{T}=y_{T}^{\prime }\right)}}{N_{T}}\;$$

where $y_{T}^{\prime }$ is the prediction label of target domain. The recognition accuracy *RC* of target domain data is the proportion of correct samples, the label predicted is same with the original, among the total test samples in one batch. Detailed experimental settings are as follows:

1. Setting 1: Take Batch 1 as source domain for model training, and test on Batch *i*, *i* = 2, 3, ..., 10.
2. Setting 2: Take Batch *i* as source domain for model training, and test on Batch (*i* + 1), *i* = 2, 3, ..., 10.

To fully demonstrate the effectiveness of the DCKT method, this paper compares the proposed DCKT method with principal component analysis (PCA), Fisher linear discriminant analysis (FLDA), SVM-RBF, SVM-comgfk algorithms without any drift suppression of data, direct standardization (DS), and the domain regularization component analysis method (DRCA) by Zhang [34].

In this paper, experiments are conducted on setting 1 and setting 2, respectively. The comparison results of the recognition accuracy for each method under setting 1 are shown in Table 2, and the bold number represents the highest accuracy rate in one batch. From the table, we can observe that the proposed DCKT method achieves the best classification performance. The average recognition accuracy reached 79.39%. At the same time, the paper also gives the parameters under the best accuracy rate obtained by using the proposed algorithm in each batch, as shown in Table 3. The accuracies of Batch 8, Batch 9, and Batch 10 are not high enough, which may be due to the fact that the presented method is under a weak assumption, i.e., the marginal probability distribution of source domain and target domain are consistent after mapping φ, then the conditional distribution will be consistent. However, the conditional distribution may not be consistent in many real-world applications. 

Under Setting 2, the *i*-th batch of data is used as the source domain to train model, and the (*i* + 1)-th batch of data is used as the target domain for testing, *i* = 1, 2, ..., 9, which used Batch (*i*)⟶Batch (*i* + 1) to indicates in this paper. The comparison results of the recognition accuracy for each method under Setting 2 are shown in Table 4. From the table, it can be seen that the recognition accuracy of the DCKT method is the highest, and the average recognition accuracy reaches 83.78%, which is 9.56% higher than the second best learning method, i.e., DRCA. At the same time, Table 5 shows the parameter values under Setting 2. The comparison results of Setting 1 and Setting 2 strongly prove the effectiveness of the proposed DCKT method.

## 5. Conclusions and Future Work

In this paper, a novel domain correction based on the kernel transformation method (DCKT) is proposed for drift compensation in an e-nose. The method is motivated from the different probability distributions of the source domain and the target domain, and the ideas of transfer learning. First, DCKT can be used for domain correction, which solves the problem of different distribution between two domains. Then the source and target domain data after domain correction can be used to train and classify with the same prediction model. The effectiveness of the proposed DCKT method is proved through experiments on the public benchmark sensor drift dataset of an e-nose.

A series of studies on sensor drift compensation have been conducted through domain correction. However, the proposed DCKT method is offline drift suppression. Therefore, achieving online drift suppression is an interesting research direction in e-noses.

## Figures and Tables

**Table 1:** Distribution of benchmark sensor drift dataset from UCSD.

Batch ID	Month	Acetone	Acetaldehyde	Ethanol	Ethylene	Ammonia	Toluene
Batch 1	1,2	90	98	83	30	70	74
Batch 2	3,4,8–10	164	334	100	109	532	5
Batch 3	11~13	365	490	216	240	275	0
Batch 4	14,15	64	43	12	30	12	0
Batch 5	16	28	40	20	46	63	0
Batch 6	17~20	514	574	110	29	606	467
Batch 7	21	649	662	360	744	630	568
Batch 8	22,23	30	30	40	33	143	18
Batch 9	24,30	61	55	100	75	78	101
Batch 10	36	600	600	600	600	600	600

**Table 2:** Recognition accuracy (%) under Experimental Setting 1.

Methods	Batch ID									Average Value
	2	3	4	5	6	7	8	9	10	
PCA_SVM_	82.40	84.80	80.12	75.13	73.57	56.16	48.64	67.45	49.14	68.60
LDA_SVM_	47.27	57.76	50.93	62.44	41.48	37.42	**68.37**	52.34	31.17	49.91
SVM-rbf	74.36	61.03	50.93	18.27	28.26	28.81	20.07	34.26	34.47	38.94
SVM-comgfk	74.47	70.15	59.78	75.09	73.99	54.59	55.88	70.23	41.85	64.00
DS	69.37	46.28	41.61	58.88	48.83	32.83	23.47	**72.55**	29.03	46.98
DRCA	89.15	**92.69**	**87.58**	**95.94**	86.52	60.25	62.24	72.34	52.00	77.63
DCKT	**90.27**	90.29	83.23	76.14	**96.26**	**75.51**	66.67	71.06	**65.06**	**79.39**

**Table 3:** Parameter values of the DCKT under Experimental Setting 1.

Batch ID	2	3	4	5	6	7	8	9	10
μ	0.001	10,000	20	1000	0.001	1000	10,000	1000	10,000
m	16	5	8	11	8	8	4	11	5

**Table 4:** Recognition accuracy (%) under Experimental Setting 2.

Methods	Batch ID									Average Value
	1⟶2	2⟶3	3⟶4	4⟶5	5⟶6	6⟶7	7⟶8	8⟶9	9⟶10	
PCA_SVM_	82.40	**98.87**	83.23	72.59	36.70	74.98	58.16	84.04	30.61	69.06
LDA_SVM_	47.27	46.72	70.81	85.28	48.87	75.15	77.21	62.77	30.25	60.48
SVM-rbf	74.36	87.83	90.06	56.35	42.52	**83.53**	**91.84**	62.98	22.64	68.01
SVM-comgfk	74.47	73.75	78.51	64.26	69.97	77.69	82.69	85.53	17.76	69.40
DS	69.37	53.59	67.08	37.56	36.30	26.57	49.66	42.55	25.78	45.38
DRCA	89.15	98.11	**95.03**	69.54	50.87	78.94	65.99	84.04	36.31	74.22
DCKT	**90.27**	91.87	90.68	**97.46**	**75.30**	78.88	75.22	**97.66**	**57.36**	**83.78**

**Table 5:** Parameter values of the DCKT under Experimental Setting 2.

Batch ID	1⟶2	2⟶3	3⟶4	4⟶5	5⟶6	6⟶7	7⟶8	8⟶9	9⟶10
μ	0.001	10,000	0.001	0.001	10,000	10,000	10,000	1000	10,000
m	16	8	32	32	7	64	8	64	17
